# TSH receptor autoantibody levels post-total thyroidectomy in Graves’ ophthalmopathy: a meta-analysis

**DOI:** 10.1007/s00423-023-03153-3

**Published:** 2023-10-23

**Authors:** Arsalan Anees, Femi E. Ayeni, Guy D. Eslick, Senarath Edirimanne

**Affiliations:** 1 Department of Surgery, Nepean Hospital, Penrith, 2750 Australia; 2 Nepean Institute of Academic Surgery, Nepean Clinical School, The University of Sydney, 62 Derby St, Penrith, NSW 2750 Australia; 3 Sydney Medical School, The University of Sydney, Sydney, Australia

**Keywords:** TSH receptor autoantibodies (TRAbs), Thyroid-stimulating hormone receptor antibodies, Graves’ ophthalmopathy, Thyroid eye disease, Total thyroidectomy, Thyroid ablation

## Abstract

**Background:**

TSH receptor autoantibodies (TRAbs) are pathognomonic for Graves’ disease and are thought to also underly the pathogenesis of Graves’ ophthalmopathy (GO). A decline in TRAb levels has been documented post-total thyroidectomy (TTx) in GO, however with conflicting correlations with disease outcomes. The aim of the study was to compare the effectiveness of TTx to other treatment modalities of Graves’ disease and examine whether the lowering of TRAbs is associated with GO improvements.

**Method:**

We searched electronic databases including Medline, Embase, Scopus, and Web of Science until 31 September 2022 using a broad range of keywords. Patients with GO undergoing TTx with measurements of both TRAbs and progression of the disease using a validated GO scoring system were included. Fourteen studies encompassing data from 1047 patients with GO met our eligibility criteria. The PRISMA guidelines were followed, and five studies had comparable data that were suitable for a meta-analysis.

**Results:**

The Cochrane Risk of Bias tool for RCTs showed low risk of bias across most domains. The pooled odds ratio showed that more patients significantly had normalized TRAb levels post-TTx as compared to other interventions (OR: 1.36, 95% CI: 1.02–1.81, *p* = 0.035). But, there was no significant difference in GO improvement post-TTx as compared with other intervention groups.

**Conclusions:**

This meta-analysis shows that TRAb levels may decline largely post-TTx, but may not predict added improvements to the progression of GO. Thus, future studies with uniform designs are required to assess the minimal significant GO improvements.

**Supplementary Information:**

The online version contains supplementary material available at 10.1007/s00423-023-03153-3.

## Introduction

Graves’ ophthalmopathy (GO) is an autoimmune disorder of the eye that is characterized by orbital soft tissue swelling, exophthalmos, and resulting visual symptoms. It is the most common extrathyroidal manifestation of Graves’ disease, which may be clinically relevant in 25–50% of patients [1]. While the usual course is benign, it may progress to compress the optic nerve to cause vision loss in 3–5% of patients with Graves’ disease [2]. The underlying pathology is thought to be linked to the shared TSH receptor antigen found in the orbital fibroblasts [3]. It is postulated that in Graves’ disease patients, TSH receptor autoantibodies (TRAbs) produced by intrathyroidal B-cells are central to the disease. These autoantibodies lead to the overstimulation of the TSH receptor on retro-ocular fibroblasts and adipocytes, resulting in orbital fat expansion and increased tissue volume [3]. Total thyroidectomy (TTx) is a well-established treatment option for the condition that aims to achieve complete removal of the thyroid gland [4]. The American Thyroid Association recommends TTx as one of the acceptable first-line treatment options for both Graves’ disease and Graves’ ophthalmopathy [5]. Moreover, it is listed as a preferred option over radioactive iodine (RAI) in moderate-to-severe or sight-threatening GO [5]. It is proposed that TTx works by removing the target tissue for autoantibodies and cause decline in TRAb post-treatment. This observation has been documented by several studies [6–9]. Previous meta-analysis that compared subtotal thyroidectomy (STx) to TTx found no difference in regression of GO with either surgical technique, suggesting that the total removal of thyroid antigens may be less relevant than previously suggested [10]. However, this review was limited by the number of studies with measurements of TRAb level to comment on the difference in decline in TRAb level post-TTx. Thus, little is known whether the decline in autoantibody post-TTx is significant and if it has an impact on GO outcomes. Therefore, in this study, we aimed to determine if the decline in TRAbs is associated with improvements in GO.

## Material and methods

### Literature search strategy

This study was conducted following the PRISMA guidelines [11]. We searched for articles across four publicly available electronic databases, including Medline, Embase, Scopus, and Web of Science. The search was limited to the English language or English translations with no limit on publication dates up to 30th September 2022. We utilized common keywords and MESH terms on Medline and adopted our search strategy for other databases (Supplementary Fig. S1). The comparator and outcome terms were omitted to avoid missing relevant studies. The search terms on Medline were as follows: “Graves’ Disease” OR “Thyroid Associated Orbitopathy” OR “Thyroid Eye Disease” OR “Graves’ Ophthalmopathy” OR “Graves Orbitopathy” AND “Thyroidectomy.” The data extraction was done in duplicate by two authors, AA and FA. We also searched manually the reference list from the eligible studies and systemic reviews to identify any additional articles.

### Inclusion criteria

We selected studies that included patients with GO undergoing TTx with measurements of both TRAb levels and progression of the disease using a validated scoring system. This included randomized controlled trials, cohort, case-control, and qualitative studies. A validated scoring system included CAS (Clinical Activity Score), NOSPECS (no physical signs or symptoms, only signs, soft tissue involvement, proptosis, extraocular muscle involvement, corneal involvement and sight loss), EUGOGO (European Group of Graves’ Orbitopathy), VISA (vision, inflammation, strabismus, appearance), and their respected variants. The diagnostic criteria for GO were defined as: (i) characteristic ocular abnormalities on clinical examination, (ii) biochemically confirmed current or past Graves’ hyperthyroidism (low TSH and high T3/T4 levels), and (iii) presence of TRAbs.

### Data extraction and quality assessment

Microsoft Excel table was used to summarize key information from the eligible studies. The information extracted from the studies included in the meta-analysis were as follows: primary author’s name, year of publication, interventions included in the study, concurrent additional treatments, country of origin of the study, age of participants, follow-up duration, GO scoring system used, the number of patients with normalized or unnormalized TRAb levels after the last follow-up post-TTx and intervention groups, and the number of patients with improved, unchanged, or worsened GO outcomes after the last follow-up post-TTx and intervention groups. Normalized TRAb levels in our study refer to a return to the baseline normal range as defined by the biochemical assay used. As a corollary, unnormalized TRAb levels refer to elevated TRAb levels. This reporting system for TRAb levels and GO outcomes was used as it allowed cross-comparison and captured the largest dataset. Similar information was extracted from studies included in the qualitative analysis. Variations in the reporting system used for TRAb levels and GO outcomes were documented where applicable.

The risk of bias assessment for all randomized controlled trials (RCTs) was performed using the Cochrane risk of bias tool for randomized trials (ROB 2) according to the Cochrane Handbook for Systematic Reviews of Interventions [12]. The risk of bias assessment for all cohort studies was performed using the Newcastle-Ottawa Scale (NOS) (Supplementary Fig. S3) [13].

### Statistical analysis

A random-effects model was used to calculate the pooled odds ratio (OR) and 95% confidence interval to compare between TTx and other interventions concerning the number of patients with normalized TRAb levels, unnormalized TRAb levels, improved GO scores, unchanged GO, and worsened GO. The heterogeneity among the studies was assessed using the *I*^2^ statistic where *I*^2^ values of 25%, 50%, and 75% were considered to indicate low, moderate, and high heterogeneity, respectively. To examine the publication bias, we used Egger’s regression model where a *p*-value of <0.05 was considered to indicate publication bias.

## Results

### Literature search

**Fig. 1:**
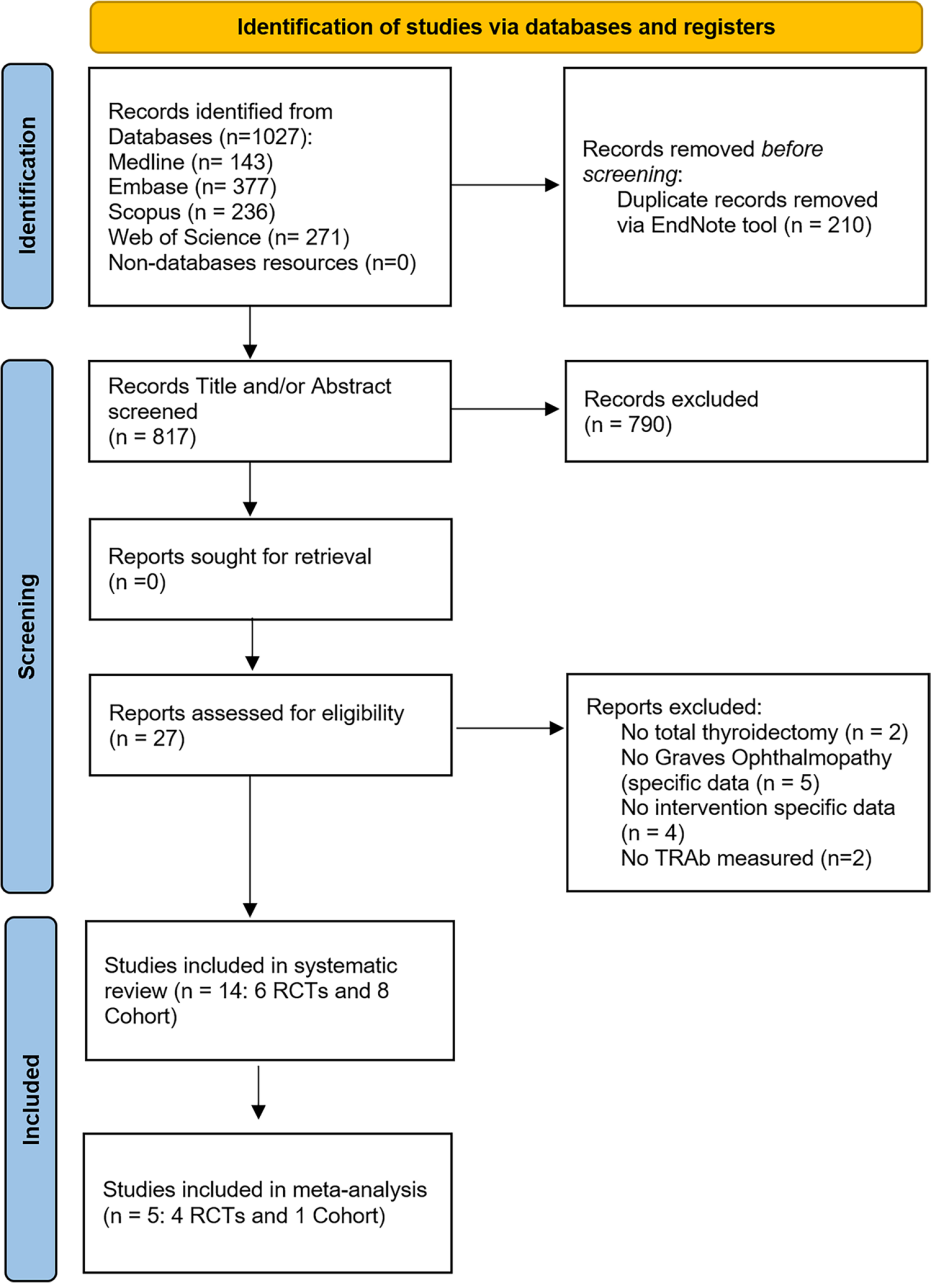
Flow diagram of study selection

The search strategy results are summarized using the PRISMA flowchart in Fig. 1. After the exclusion of duplicates, a total of 817 articles were identified. Of these, 790 were excluded based on their title and abstract due to their ineligibility with the inclusion criteria. A total of 27 articles underwent full-text examination, of which 13 articles were excluded. The reasons for exclusion were as follows: no TTx performed (*n* = 2), no GO-specific data (*n* = 5), no intervention-specific data (*n* = 4), and no TRAb levels measured (*n* = 2). Therefore, 14 articles (six RCTs, eight cohort studies) encompassing data from 1047 patients with GO were included in this systematic review [7, 9, 14–24]. Furthermore, five of these articles (four RCTs, one cohort study) had comparable data that were suitable for a meta-analysis [14–18].

### Studies included

The characteristics of all 14 studies included in the review are summarized in Table 1. Most studies were conducted in European population groups while Catz et al. examined North American patients, Erdogan et al. and Nart et al. examined Turkish patients [7, 23]. Most studies included predominately adult patients and sample sizes varied from 32 to 200 patients. The follow-up duration post-surgery ranged from as early as 21 days for some patients in the study by Nart et al. to 9 years in the study by Catz et al. [7, 20]. One study compared TTx to RAI, four included STx comparison groups, four included total thyroid ablation groups (near or total thyroidectomy followed by RAI), three included ATD (antithyroid drugs), and three studies had no comparison groups.

**Table 1:** Characteristics of the 14 eligible studies

Author, year of publication	Study type	Intervention	Concurrent additional treatment	Country	Study size	Age (years)	Follow-up duration	GO scoring system	TRAbs (TTx)	TRAb (intervention)	GO (TTx)	GO (intervention)
Studies included in meta-analysis												
Kautbally et al., 2012	Retrospective cohort study	TTx vs. RAI	ATD	Belgium	80	TTx: 38.8 ± 14.4 SD RAI 43.2 ± 16.0 SD	3 years	Ophthalmologist reports	Normal: 33/40 Unnormalized: 7/40	Normal: 16/40 Unnormalized: 24/40	Improved: 10/40 Unchanged: 29/40 Worsened: 1/40	Improved: 2/40 Unchanged: 33/40 Worsened: 5/40
Barcyznski et al., 2012	Randomized controlled trial	TTx vs. STx	None mentioned/recorded	Poland	200	TTx: 46.2 (95% CI 43.5–48.9) STx: 45.5 (95% CI 43.1–47.9)	5 years	NOSPECS-TES	Normal: 74/96 Unnormalized: 22/96	Normal: 48/95 Unnormalized: 47/95	Improved: 85/96 Unchanged: 4/96 Worsened: 7/96	Improved: 81/95 Unchanged: 5/95 Worsened: 9/95
Witte et al., 2000	Randomized controlled trial	TTx vs. STx	ATD, beta-adrenergic drugs, thyroid hormones or combination	Germany	150	TTx: median 38 (range 21–68) BST: median 36 (range 16–73) Dunhill: median 41 (range 12–66)	18–58 months	Modified ATA scale	Normal: 30/47 Unnormalized: 6/47	Normal: 54/103 Unnormalized: 17/103	Improved: 22/31 Unchanged: 7/31 Worsened: 2/31	Improved: 41/56 Unchanged: 11/56 Worsened: 4/56
Moleti et al., 2014	Randomized controlled trial	TTx vs. thyroid ablation	ATD, IV steroids post-surgery	Italy	40	TTx: median 47 (range 19–65) Thyroid ablation: median 46 (range 19–61)	1 year	EUGOGO	Normal: 12/20 Unnormalized: 8/20	Normal: 13/20 Unnormalized: 7/20	Improved: 4/20 Unchanged: 11/20 Worsened: 5/20	Improved: 14/20 Unchanged: 6/20 Worsened: 0/20
Leo et al., 2012	Randomized controlled trial	TTx vs. thyroid ablation	IV steroids, radiotherapy, orbital decompression, muscle surgery, or eyelid surgery on case per case basis	Italy	60	TTx: 45.7 ± 8.8 SD Thyroid ablation: 46.4 ± 11.2 SD	Mean 88.0 ± 17.7 months	CAS	Normal: 18/25 Unnormalized: 7/25	Normal: 21/27 Unnormalized: 6/27	Improved: 10/25 Unchanged: 13/25 Worsened: 2/25	Improved: 13/27 Unchanged: 14/27 Worsened: 0/27
Studies included in qualitative analysis												
De Bellis et al., 2011	Prospective cohort study	TTx vs. thyroid ablation vs. ATD	None	Italy	60	TTx: 36.10 ± 4.93 SD Thyroid ablation: 37.05 ± 5.0 SD ATD: 37.02 ± 5.0 SD	2 years	CAS	1.2 ± 0.54 (from baseline of 9.08 ± 2.5) * (*p* = 0.001)	TTX+RAI: 0.9 ± 1.1 (from baseline 8.9 ± 2.7) * (*p* < 0.04) ATD: 4.1 ± 2.7 (from baseline 9.48 ± 3.38) * (*p* < 0.04)	1.7 ± 0.75 (from baseline 4.5 ± 0.5) * (*p* = 0.001)	TTX + RAI: 1.1 ± 02 (from baseline 4.2 ± 0.45) * (*p* < 0.04) ATD: 1.8 ± 0.8 (from baseline 4.2 ± 0.4) * (*p* < 0.001)
Nart et al., 2008	Retrospective cohort study	TTx	None	Turkey	35	Median 35 (range 16–72)	Mean 422 days (range 21–1306 days)	Modified NOSPECS	3.4 U/L from baseline 33.8 * (*p* < 0.0000)	-	1.52 from baseline 2.84 * (*p* < 0.0000)	-
Domoslawski et al., 2007	Retrospective cohort study	TTx	ATD	Poland	69	31	18 months	ATA scale	1.1 (±0.3) from 13.3 (±13.1) before surgery; nonsignifigant	-	Improved: 17/42 Unchanged or worsened: 25/42	-
Catz et al., 1969	Retrospective cohort study	TTx	ATD, lugol solution and thyroid hormones	USA	82	Age group < 20: 15 Age group 20–40s: 47 Age group 40–60s: 20	Up to 9 years	Classification of Hamilton, Schultz, and De Gowin	Normal: 66/70 Unnormalized: 4/70	-	Improved: 66/70 Unchanged: 4/70 Worsened: 0/70	-
Myer zu Horste et al., 2016	Retrospective cohort study	TTx vs. ATD	ATD	Germany	92	TTx: median 48.8 (range 20–68) ATD: median 50.8 (range 27–78)	6 months	CAS	5.2 (0.1–65.2) from baseline 18.6 (0.9–123.7)	3.2 (0.1–53.1) from baseline 12.8 (0.4–97.5)	2.1 ± 1.3 from baseline 5.9 ± 1.5	2.8 ± 1.5 from baseline 5.7 ± 1.5
Konturek et al., 2008	Retrospective cohort study	TTx vs. STx	ATD, beta blockers, and IV steroids	Poland	61	45.5 ± 12	12 months	Donaldson ophthalmopathy index	23 absent (<1 IU/L) 8 doubtful (1–2 IU/L)	12 absent (<1 IU/L) 1 present (>2 IU/L) 10 doubtful (1–2 IU/L)	3.24 from baseline 6.49 * (*p* < 0.001)	BST: 5.67 from baseline 2.7 (n.s.) Dunhill: 4.91 from baseline 2.41 (n.s.)
Erdogan et al., 2016	Randomized controlled trial	TTx vs. ATD	ATD and IV steroids	Turkey	42	TTx: 44 ± 8.7 ATD: 35 ± 12.9	36 months	CAS	13 from baseline 80 IU/L (*p* < 0.01)	16 from baseline 54 (n.s.)	1 from baseline 4 (*p* < 0.001)	0 from baseline 3 (*p* < 0.001)
Jarhult et al., 2005	Randomized controlled trial	TTx vs. STx	None mentioned/recorded	Poland	44	TTx: median 44 (range 21–62) STx: median 42 (range 24–69)	3 years	Total activity score	Median TRAb 7 U/L from baseline median 56 U/L (normal <5) Undetectable in 9 (41%)	Median TRAb 5 U/L from baseline median 60 U/L (normal < 5) Undetectable in 12 (57%)	Median 1.0 (0–8)	Median 1.0 (0–4)
Lanzolla et al., 2021	Retrospective cohort study	Total thyroid ablation vs. TTx	IV steroids	Italy	32	TTx: 47.2 ± 11.3 (range 19–64) Thyroid ablation: 48 ± 9.7 (range 26–60)	48 weeks	CAS	-	-	69.2% improvement (*p* = 0.347)	52.6% improvement (*p* = 0.347)

*ATD*, antithyroid drugs; *GO*, Graves’ ophthalmopathy; *IV*, intravenous; *RAI*, radioactive iodine ablation; *STx*, subtotal thyroidectomy; *TRAbs*, thyroid receptor autoantibodies; *TTx*, total thyroidectomy

Five studies were included in the meta-analyses and comprised a total of 530 patients with GO. The meta-analysis included one study with the RAI group, two studies with STx groups, and two studies with total thyroid ablation. These studies were conducted in European population groups and included predominately adult patients. The follow-up duration ranged from as early as 18 months in the study conducted by Witte et al. to 5 years post-surgery in the study by Barcyznski et al. [15, 16].

### Risk of bias

The risk of bias assessment of the RCTs and cohort studies are presented in Supplementary Fig. 2A and Supplementary Table S1, respectively. The risk of bias in the RCTs included in our study indicated that while there was a low risk of bias across most of the domains of the Cochrane risk of bias tool, there was overall some level of concern (Supplementary Fig. 2B). This mostly stemmed from the lack of blinding in most of the RCTs included in the review. The RCTs by Witte et al., Moleti et al., Leo et al., and Jarhult et al. were single-blinded as either the investigators or patients were aware of the procedure. The RCT of 200 patients comparing TTx to STx by Barcyznski et al. was the only RCT that was double-blinded as both investigators and ophthalmologists were masked to the group assignment [15]. The RCT of 42 patients by Erdogan et al. did not account for blinding in their analysis [23]. However, this study was not included in our meta-analysis since it did not have comparable GO data. The risk of bias in the cohort studies included in our review was assessed using the Newcastle-Ottawa Scale. All studies received a score of above 5, and the only cohort study included in our meta-analysis, Kautbally et al., received a score of 8, indicating a low risk of bias (Supplementary Table S1) [14].

### The decline in TRAbs levels

**Fig. 2:**
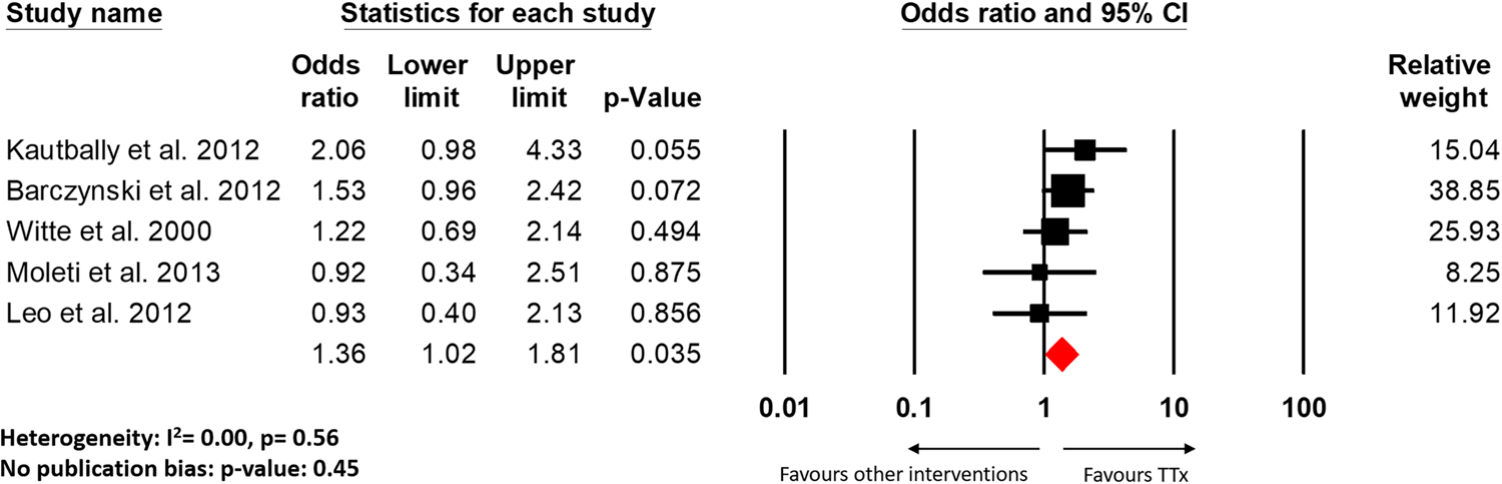
Forest plot for the comparison between TTx and other interventions on the number of patients with normalized TRAb levels after procedure

All studies included in the review showed a decline in TRAb levels post-TTx, and a statistically significant decline was documented by seven studies [7, 9, 18, 19, 21–23]. Five studies (four RCTs and one cohort study) that had a comparison group and also made qualitative assessments in regard to the normalization of TRAbs were included in our meta-analysis [14–18]. As shown in Fig. 2, while individually no study demonstrated a significant effect, the effective pooled data suggest that TRAb levels were significantly normalized after TTx as compared to other intervention groups (OR: 1.36, 95% CI: 1.02–1.81, *p* = 0.035). Similarly, significantly fewer patients had unnormalized TRAb levels post-TTx as compared to other intervention groups (OR: 0.60, 95% CI: 0.37–0.99, *p* = 0.046, Supplementary Fig. 6A). However, we found discrepancies when compared to the results of the studies that were included in this systematic review. The retrospective cohort study of 61 patients by Konturek et al. and an RCT of 42 patients by Erdogan et al. did find a significant reduction in TRAbs levels post-TTx as compared to STx and ATD, respectively [14, 15]. But De Bellis et al., Myer Zu Horste et al., and Jarhult et al. respectively found no difference when compared to thyroid ablation, ATDs, and STx, respectively [9, 21, 24].

### Improvement in Graves’ ophthalmopathy score

**Fig. 3:**
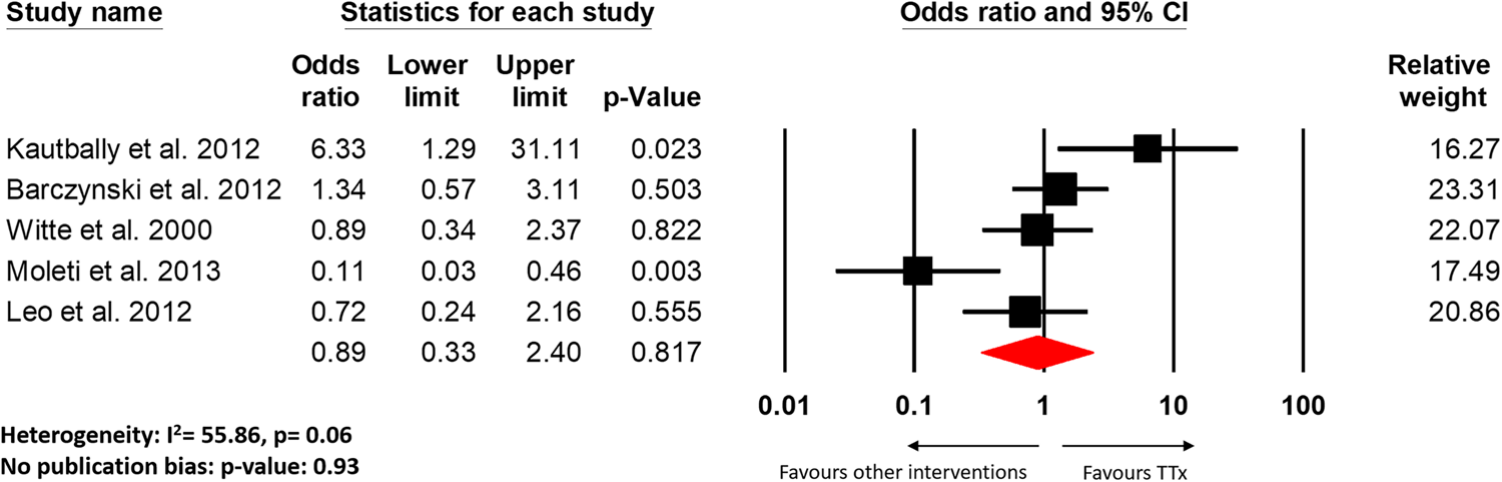
Forest plot for the comparison between TTx and other interventions on the number of patients with improved GO outcomes after procedure

**Fig. 4:**
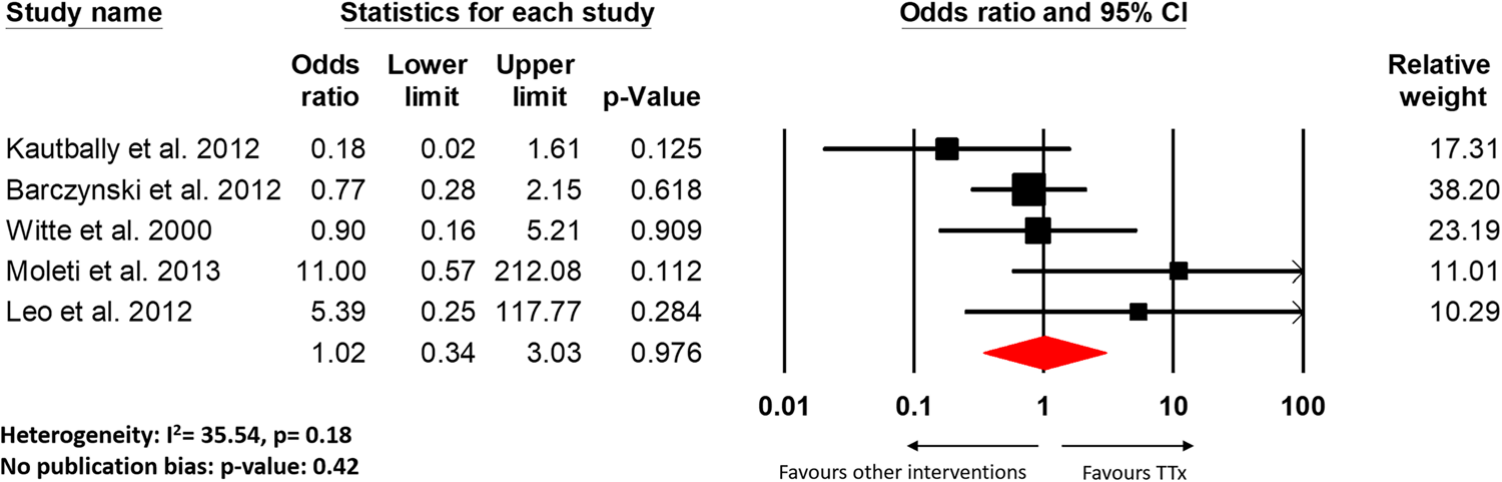
Forest plot for the comparison between TTx and other interventions on the number of patients with worsened GO outcomes after procedure

All studies included in the review showed improvement in GO outcomes post-TTx, but significant improvement was documented by five studies [7, 9, 21–23]. These five studies (four RCTs and one cohort study) had a comparison group and also made qualitative assessments in regard to GO progression, hence, were included in our meta-analysis [14–18]. There was also no significant differences found in improvement, worsening, or unchanging outcomes of GO in post-TTx as compared with other intervention groups (according to Figures 3, 4 and Supplementary Fig. 6B). This finding was consistent with the results of all of the individual studies included in the meta-analysis. Moreover, no significant difference in GO outcomes was also noted between TTx and other interventions in all but two studies included in this review [17, 21]. In the single-blinded RCT of 40 patients, Moleti et al. found that GO outcomes improved significantly following total thyroid ablation as compared to the TTx alone [17]. Similarly, the retrospective cohort study of 92 patients by Myer Zu Horste et al. showed that TTx improved the outcome of GO significantly as compared to ATD alone [21].

## Discussion

### TRAb levels post-TTx

Our results suggest that TRAb levels were significantly normalized after TTx as compared to other intervention groups (OR: 1.36, 95% CI: 1.02–1.81, *p* = 0.035, Fig. 2). This finding was consistent with Kautbally et al. and Barcyznski et al. which showed a significant reduction in TRAb levels post-TTx as compared to RAI and STx, respectively [14, 15]. But we also found that there was no significant difference in the outcomes of GO post-TTx as compared to other intervention groups (Figs. 3, 4, Supplementary Fig. 6B). These results indicate that while TRAb levels may undergo decline more post-TTx, but their is no evidence it offered any added improvements to the progression of GO. The decline in TRAb levels post-TTx has been documented by previous studies, however with conflicting correlations to improvements in the GO outcomes [6–9].

To the best of our knowledge, we understand that this is the first meta-analysis to demonstrate a significant decline in the TRAb level post-TTx and with no clinical correlation with GO outcomes. There may be several explanations for this finding, which include variability of GO scoring systems and TRAb assays used, independent variables affecting GO outcomes, alternative disease mechanisms, and limitations of our current study.

### GO scoring systems and TRAb assays

There were technical challenges in this analysis due to certain nuances in the GO scoring systems and TRAb assays used in the studies. The instruments for GO scoring systems differ in the amount of objective and subjective data that are collected from the patients. The CAS and EUGOGO classifications are mainly based on objective data and are considered to be good predictors of disease, as compared to GO quality of life questionnaires, which have been found to show only a moderate correlation with disease severity [25–27]. Moreover, the NOSPECS grading system only measures disease severity but not activity unlike the newer classifications systems such as VISA and EUGOGO [7]. While most of the studies included in our analysis used a 2nd-generation TRAb immunoassay, they do not differentiate between the stimulating (TSI) and blocking antibodies (TBII) subtypes of TRAbs. This is important to note as TSIs are known to provide a stronger positive correlation to GO severity [28–30].

### Independent variables affecting GO outcomes

TTx is preferred over ATD and RAI for more severe GO cases and those with thyrotoxicosis and a large goiter size [5]. Hence, differences in GO severity could impact the results seen when comparing TTx to other treatment options. More severe GO and higher thyrotoxicosis are also associated with higher TRAb levels and therefore are more likely to cause persistence after treatment [31]. The smoking status of the patient is also another independent factor associated with higher TRAb levels and worse clinical outcomes [32]. Moreover, there may be a timepoint variability to the results seen when comparing surgery to RAI ablation. It has been demonstrated by studies examining the course of TRAb levels that RAI ablation results in a temporary surge of antibodies after treatment followed by a gradual decline [14, 30, 33–35]. While this may be related to the dose of RAI delivered, Kautbally et al. observed a marked rise in TSI levels over the first 6 months followed by a gradual decrease, and eventual normalization of TSI levels at 18 months [13].

### Alternative disease mechanisms

The TRAb overstimulation of the orbital fibroblast TSH receptor model is currently the most well-accepted disease mechanism underlying GO progression [3]; however, new insights into the pathophysiology of GO have implicated the role of insulin-like growth factor 1 (IGF1) [36]. Along with the TSH receptor, IGF1 receptor expression in orbital fibroblasts is also increased in GO [37, 38]. Evidence from in vitro studies suggests that GO results from the stimulation of the IGF1 receptor on orbital fibroblasts with a possible synergistic interaction between TRAbs and IGR1 in increasing orbital fat expansion [36, 39]. Further evidence of this crosstalk between TRAbs and IGF was demonstrated by Krieger et al. The study showed that M22, a stimulating TRAb, which did not bind the IGF1 receptor, was also inhibited by the IGF1 receptor antagonists [40]. Further elucidation of this signalling pathway has led researchers to trial various immunosuppressive and biological agents with varying levels of success [39]. Future developments in this area could lead to new non-surgical treatment options in GO.

### Limitations of our study

Our analysis was limited by the small sample sizes of each study and a few robust RCTs with qualitative analysis of TRAb levels and GO outcomes in patients after TTx. There were nine studies not included in our meta-analysis which all showed conflicting correlations between TRAb levels and GO outcomes [7, 9, 19–24, 41].

While we do demonstrate low statistical heterogeneity in our analysis, there may still be clinical heterogeneity in our study when comparing TTx to other intervention groups which include a combination of surgical and non-surgical treatments. This was particularly evident in studies that included TTx in both control and treatment arms such as those which compared TTx to thyroid ablation. A subgroup analysis was not possible in this review due to the limited studies measuring TRAb levels and the differences in the reporting of outcomes. Future studies in this area could perform a subgroup analysis to compare the decline in TRAb levels after TTx to other surgical or non-surgical interventions. Going forward, being able to quantify the amount of TRAb and how its decline correlates with the outcome of Graves’s opthalmopathy would aid in its diagnostic applicability and management. This is an important implication to consider as TTx carries a risk of adverse effects such as permanent hypoparathyroidism or post-operative complications such as damage to the recurrent laryngeal nerve [10, 42].

## Conclusion

We found that significantly more patients had normalized TRAb levels post-TTx as compared to other interventions. However, there was no significant difference in the outcome and progression of GO post-TTx as compared with other intervention groups. These results suggest that while TRAb levels decline more post-TTx, they may not predict added improvements to GO progression.

### Supplementary information

ESM 1: Figures S1-S6 and Table S1 (DOCX 1192 kb)

## Data Availability

Original data generated and analyzed during this study are included in this published article or in the data repositories listed in the References.
