# Supplementary material for: TSH receptor autoantibody levels post-total thyroidectomy in Graves’ ophthalmopathy: a meta-analysis
Source: Langenbecks Arch Surg. 2023 Oct 23;408(1):415. doi: 10.1007/s00423-023-03153-3 (PMC10593610; doi:10.1007/s00423-023-03153-3)
Supplement: Supplementary file 1 — Figures S1-S6 and Table S1 (DOCX 1192 kb) [file 423_2023_3153_MOESM1_ESM.docx]

**Supplementary Figures**


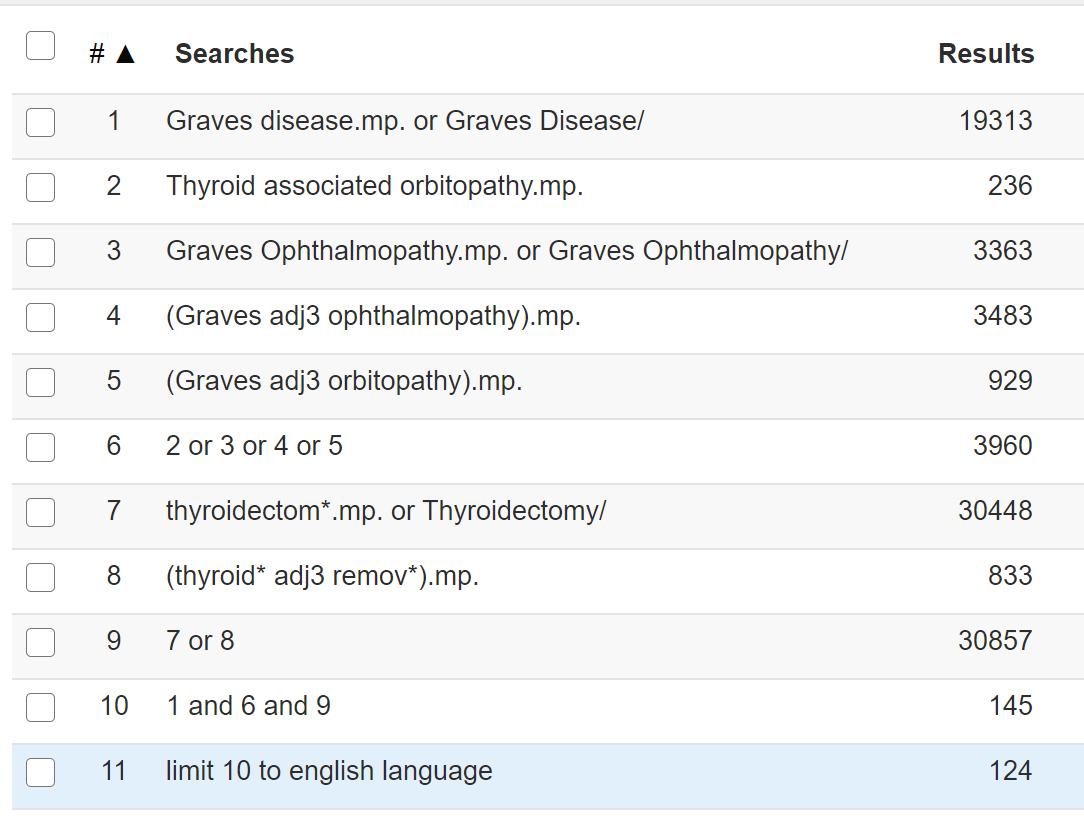


Supplementary Fig S1. A sample of the search strategy used for Medline, employing a combination of common keywords and MESH terms.

A


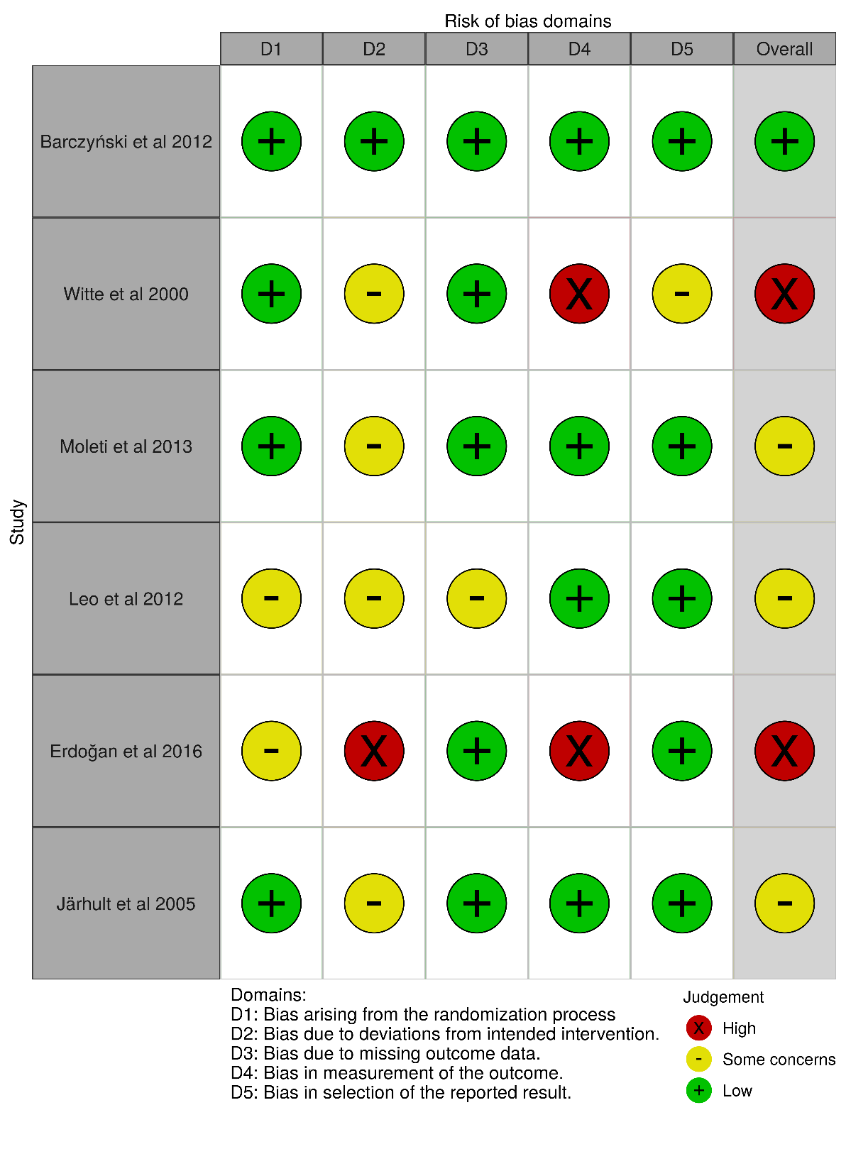


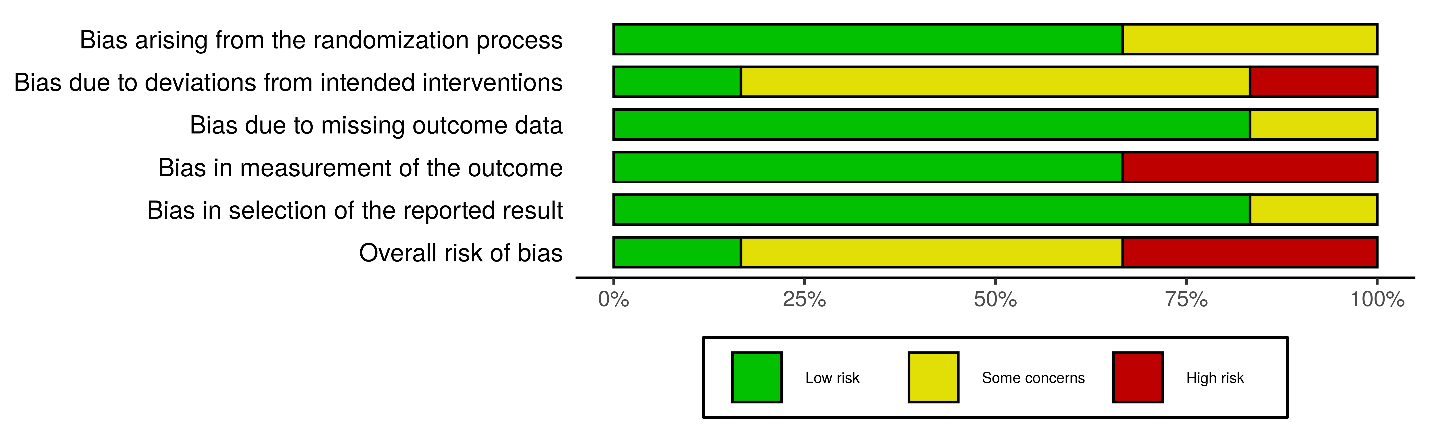
B

Supplementary Fig S2. Cochrane risk of Bias: (A) bias assessment for all randomized controlled trials included in the review. (B) summary graph illustrating risk of bias.


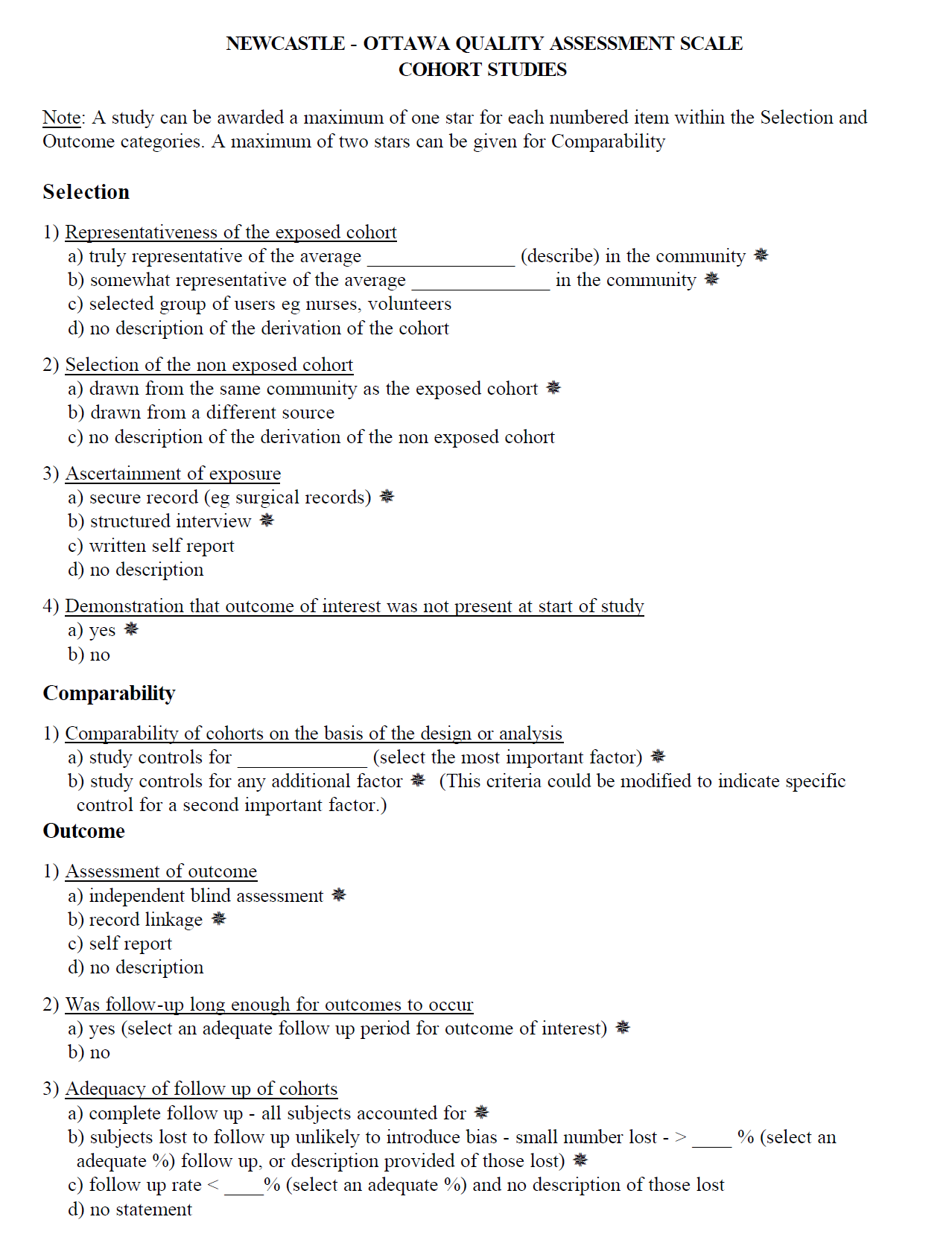


Supplementary Fig S3. NOS assessment form for cohort studies. For comparability, 2 stars were given if the studies accounted for differences in baseline age, sex, TRAb antibodies and GO severity between the groups.

P=0.25

A

P=0.25

B

C

P=0.50

A

Supplementary Fig S4. Funnel plot for evaluating publication bias of the meta-analysis for the (A) normalized TRAb levels; (B) unnormalized TRAb levels and (C) improved GO outcomes.

A

P=0.99

B

P=0.18

Supplementary Fig S5. Funnel plot for evaluating publication bias of the meta-analysis for the (A) unchanged GO outcomes; (B) worsened GO outcomes.


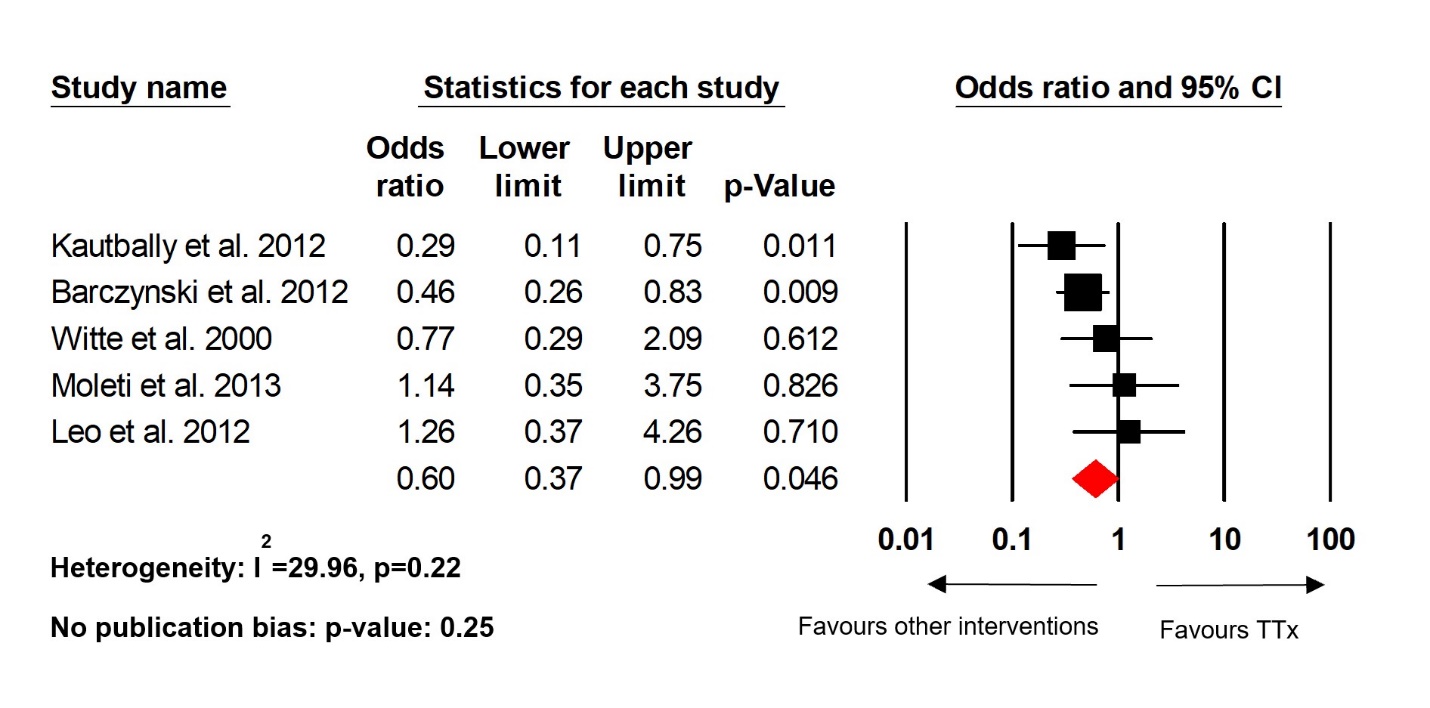


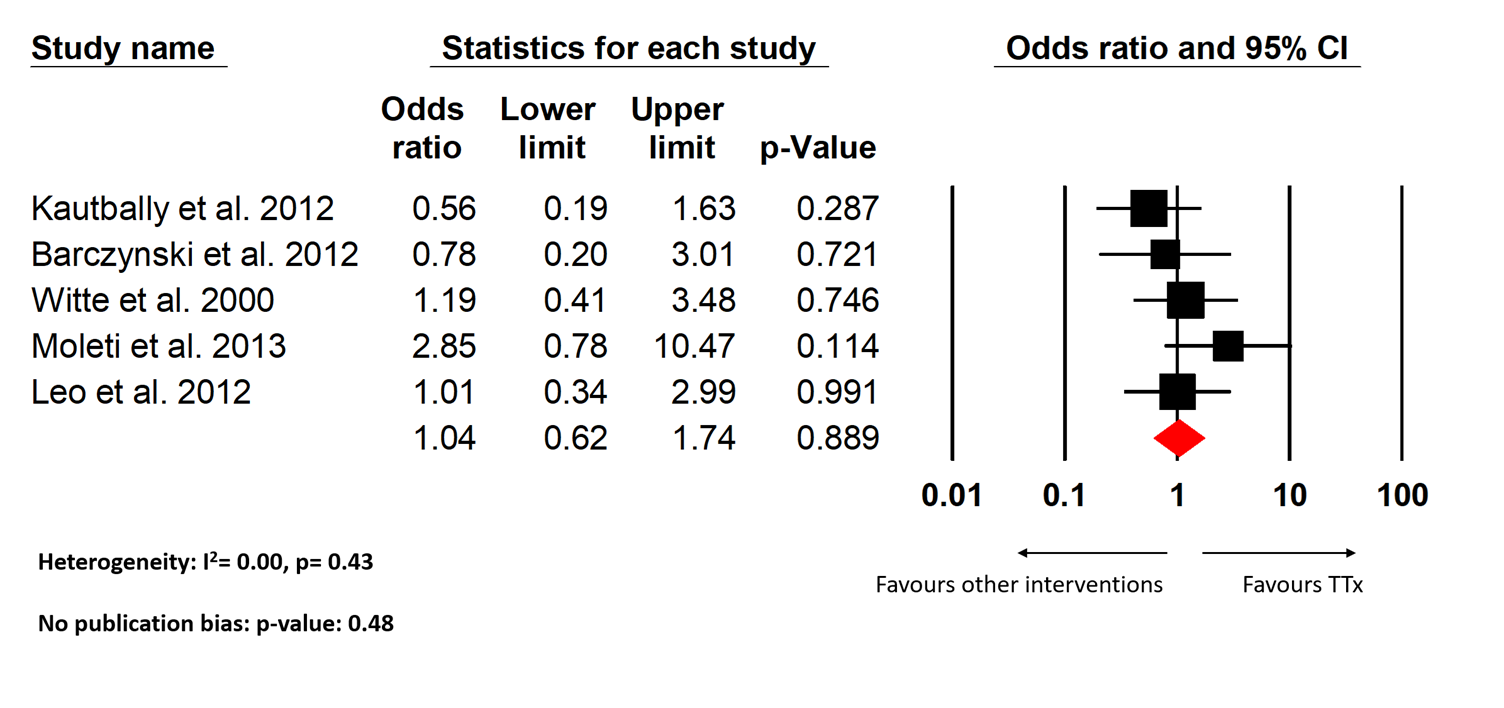


Supplementary Fig S6. Forest plot for the comparison between TTx and other interventions on the number of patients with: (**A**) unnormalized TRAb levels after procedure; and (**B**) unchanged GO outcomes after procedure.

**Supplementary Tables**

Supplementary Table S1: Newcastle-Ottawa (NOS) scores for cohort studies

| NOS Scale | Kautbally et al 2012 | De Bellis et al 2011 | Nart et al 2008 | Domoslawski et al 2007 | Catz et al 1967 | Myer Horste et al 2016 | Konturek et al 2008 |
| --- | --- | --- | --- | --- | --- | --- | --- |
| A- selection (maximum of 4 *) |  |  |  |  |  |  |  |
| 1. Representativeness of the Exposed Cohort | * | * | * | * | * | * | * |
| 2. Selection of the Non-Exposed Cohort | * | * | 0 | 0 | 0 | * | * |
| 3. Ascertainment of Exposure | * | * | 0 | 0 | 0 | * | * |
| 4. Demonstration That Outcome of interest was not present at start of study | * | * | * | * | * | * | * |
| B- Comparability (maximum of 2 * ) |  |  |  |  |  |  |  |
| 5. Comparability of Cohorts on the Basis of the Design or Analysis | * | ** | 0 | 0 | 0 | * | 0 |
| C- Outcome (Maximum of 3 *) |  |  |  |  |  |  |  |
| 6. Assessment of Outcome | * | * | * | * | * | * | * |
| 7. Was follow-up long enough for outcomes to occur | * | * | * | * | * | * | * |
| 8. Adequeacy of follow-up of cohorts | * | * | * | * | * | * | * |
| Total (Maximum of 9 *) | 8 | 9 | 5 | 5 | 5 | 9 | 7 |

NOS = Newcastle-Ottawa Scale, “*” corresponds to a point in the NOS criteria while “0” corresponds to no point in the NOS criteria, “**” was given if study accounted for baseline age, sex, TRAb antibodies and GO severity.
